# An Unusual Presentation of Crohn’s Disease and Primary Sclerosing Cholangitis

**DOI:** 10.7759/cureus.22797

**Published:** 2022-03-03

**Authors:** Lakmal S Ekanayake, Ammar Ahmad, Nicole Weyer, Sangeeta Agrawal

**Affiliations:** 1 Internal Medicine, Wright State University Boonshoft School of Medicine, Dayton, USA; 2 Genetics, Furman University, Greenville, USA; 3 Internal Medicine - Gastroenterology, Dayton VA Medical Center, Wright State University, Dayton, USA

**Keywords:** ulcerative colitis (uc), gastroenterology, primary sclerosing cholangitis, crohn’s disease (cd), inflammatory bowel disease

## Abstract

Crohn’s disease (CD) and ulcerative colitis (UC) are chronic inflammatory gastrointestinal bowel disorders that may affect any part of the alimentary tract. Classically, these two diseases have hallmark associations, such as UC with primary sclerosing cholangitis (PSC). We present a case of a healthy 21-year-old white female with concomitant CD and PSC, complicated by a biliary stricture requiring stent placement. We discuss shared risk factors, disease pathogenesis, and hallmark pathological associations. To the best of our knowledge, there are a limited number of reported cases that demonstrate the dual phenotype of CD and PSC.

## Introduction

Crohn’s disease (CD) and ulcerative colitis (UC) belong to the family of inflammatory bowel disease (IBD). Classically, UC has been associated with primary sclerosing cholangitis (PSC). A rare and unique manifestation is the concomitant presentation of CD and PSC. The duality of CD-PSC demonstrates unique complications including biliary stricture, liver cirrhosis, and nutritional deficiencies.

At the molecular level, CD is a predominately pro-inflammatory, chronic intestinal pathology. Recent evidence has supported a theory of inappropriate response via intestinal microbes in a susceptible host [1]. Pathological findings include transmural inflammation, abscess formation, and fistula formation [2].

In contrast, there has been significant evidence showing UC associated with the region of chromosome 12q15, which includes interferon-gamma and interleukin 26 [3]. Key pathological findings of UC include inflammation primarily confined to the mucosa and submucosa with superficial and deep ulcerations with crypt abscesses. Anatomically, UC affects primarily the colon and rectum. Importantly, the strongest association to UC is the development of PSC.

Clinically, the diagnosis of IBD is life-changing, yet many times the clinical picture is complicated by generalized symptoms and non-specific complaints making management complex and individualized.

## Case presentation

**Figure 1:**
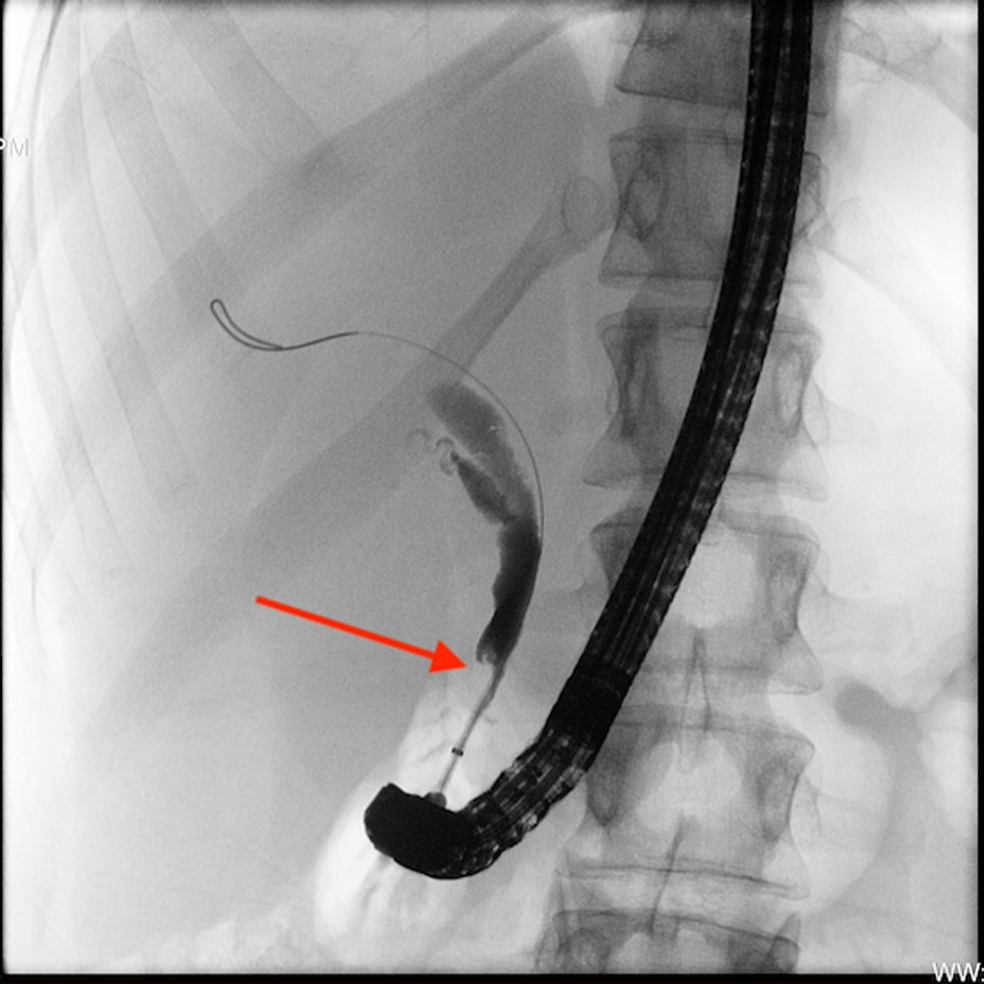
Balloon cholangiogram. A stent placed into the main bile duct bridging the distal common bile duct stricture.

A 21-year-old white female presented to the hospital for planned endoscopic retrograde cholangiopancreatography (ERCP) due to suspected blockage of the gallbladder secondary to an unknown etiology. Previous imaging studies suggested bile duct dilatation and a possible stricture of the distal common bile duct (CBD). Hepatobiliary iminodiacetic acid (HIDA) scan five days prior was unremarkable. Symptoms included right upper quadrant (RUQ) abdominal pain, which persisted for several months. Past medical history included PSC, CD, gastroesophageal reflux disease, and chronic migraines. Surgical history included several colonoscopies and upper endoscopies. Current medications were amitriptyline, lansoprazole, loperamide, and mesalamine. The patient had no significant social history and, importantly, she did not have any direct family or first-degree relatives with IBD, CD, UC, PSC, or primary biliary cirrhosis. ERCP findings showed short-segment bile duct strictures with dilated upstream extrahepatic ducts. The CBD stricture was dilated to 4 mm, and a biliary stent was placed into the main bile duct bridging the distal CBD stricture (Figure 1). Based on the endoscopic findings, repeat ERCP was completed four weeks after initial stent placement. At that time, areas of multifocal stenosis and post-stenotic dilation were seen in the CBD and common hepatic duct. Two stents were placed. A third ERCP was completed four weeks later, and stents were removed. At one-week follow-up, the patient reported that her RUQ pain had improved. Abdominal ultrasound two weeks post stent removal showed stable intrahepatic and extrahepatic biliary ductal dilatation, with CBD measuring up to 8 mm in its largest diameter.

## Discussion

PSC is a chronic liver disease, where localized inflammation and fibrosis can lead to multiple fibrotic strictures along the biliary tree, causing strictures and complete bile outflow obstruction. PSC is divided into several subtypes, which affect various portions of the biliary system. The most classic presentation affects over 90% of the entire biliary tree [4]. Secondly, a small minority of patients have PSC, which only affects the small intrahepatic bile ducts [4].

Mechanistically, there has been a multitude of theories. One of the leading pathological stepwise mechanisms is progressive concentric fibrosis around the bile ducts. This progressive concentric fibrosis is thought to be influenced by a host of genetic and environmental risk factors [4]. As mentioned, PSC is strongly associated with IBD, specifically UC, in addition to the increased likelihood of the formation of certain cancers such as cholangiocarcinoma. PSC presenting with CD is a rare association, with less than 5% of CD patients being diagnosed with associated PSC [5]. UC with concomitant PSC is more classically diagnosed, with an incidence of approximately 7.5% [5].

Clinically, patients with abnormally functioning liver enzymes have hepatomegaly, splenomegaly, painful abdomen to palpation, pruritus, and jaundice on the physical exam [6]. In addition, patients may have night sweats, fatigue, and weight loss. Diagnosis can be achieved by increased serum alkaline phosphatase for greater than six months in addition to endoscopic findings via cholangiography of strictures within the bile ducts [4]. Secondly, diagnosis can be achieved using a cholangiogram via magnetic resonance cholangiopancreatography or a percutaneous liver biopsy [6].

Treatment of patients with PSC includes ursodeoxycholic acid, which has not been approved by the American Association for the Study of Liver Diseases but has been approved by the European Association for the Study of the Liver. Due to the progressive fibrotic nature of this disease, nearly half of these patients will require a liver transplant [7].

## Conclusions

CD presenting with concomitant PSC in a female patient is rare. Various complications may present including biliary strictures, liver cirrhosis, and nutritional deficiencies. Treatment includes biological therapy, immunosuppressant drugs, and liver transplantation. This case provides evidence of a rare complication from IBD and associated treatment modalities.
